# Sense of agency predicts severity of moral judgments

**DOI:** 10.3389/fpsyg.2022.1070742

**Published:** 2023-02-02

**Authors:** Chiara Spaccasassi, Kamela Cenka, Stella Petkovic, Alessio Avenanti

**Affiliations:** ^1^Centre for Studies and Research in Cognitive Neuroscience, Department of Psychology, Alma Mater Studiorum Università di Bologna, Cesena, Italy; ^2^"Sapienza" University of Rome and CLN2S@SAPIENZA, Istituto Italiano di Tecnologia, Rome, Italy; ^3^Centro de Investigación en Neuropsicología y Neurosciencias Cognitivas, Universidad Católica Del Maule, Talca, Chile

**Keywords:** sense of agency, intentional binding, morality, pain, accidental harm

## Abstract

Sense of Agency (SoA) refers to the awareness of being the agent of our own actions. A key feature of SoA relies on the perceived temporal compression between our own actions and their sensory consequences, a phenomenon known as “Intentional Binding.” Prior studies have linked SoA to the sense of responsibility for our own actions. However, it is unclear whether SoA predicts the way we judge the actions of others – including judgments of morally wrong actions like harming others. To address this issue, we ran an on-line pilot experiment where participants underwent two different tasks designed to tap into SoA and moral cognition. SoA was measured using the Intentional Binding task which allowed us to obtain both implicit (Intentional Binding) and explicit (Agency Rating) measures of SoA. Moral cognition was assessed by asking the same participants to evaluate videoclips where an agent could deliberately or inadvertently cause suffering to a victim (Intentional vs. Accidental Harm) compared with Neutral scenarios. Results showed a significant relation between both implicit and explicit measures of SoA and moral evaluation of the Accidental Harm scenarios, with stronger SoA predicting stricter moral judgments. These findings suggest that our capacity to feel in control of our actions predicts the way we judge others’ actions, with stronger feelings of responsibility over our own actions predicting the severity of our moral evaluations of other actions. This was particularly true in ambiguous scenarios characterized by an incongruency between an apparently innocent intention and a negative action outcome.

## 1. Introduction

In our everyday life, we physically interact with the environment by changing the course of external events. Under normal conditions, we are consciously aware of being the agents of our own actions by assuming responsibility for their effects. This phenomenon is called “Sense of Agency” (SoA; Haggard and Chambon, 2012; Haggard, 2017).

In recent years, several studies have characterized the features of SoA and endorsed the hypothesis that it consists of both prospective and retrospective cognitive evaluations of executed actions and their consequences (e.g., Gallagher, 2012; Chambon et al., 2014; Haggard, 2017). Specifically, before performing an action, the agent would first select which action to perform from a variety of possibilities and, in a second step, a prediction of its future output would be envisaged (the prospective component). As soon as the action has been performed, the feedback from its outcome is available to the agent, allowing comparison between the predicted and actual signals (the retrospective component). The final output of this comparison, which is called the “prediction error,” is essential for defining the strength of SoA: people will feel more in control of their actions if a prediction error does not occur (or it is negligible), compared to situations where there are substantial incongruencies between the predicted and actual outcomes (Fletcher and Frith, 2009; Carruthers, 2012; Seth et al., 2012; Haggard, 2017). Thus, temporality plays a pivotal role in this “comparator model” of SoA: events occurring too early or too late in time compared to the performed action are not considered to be linked outcomes (Frith et al., 2000; Ruess et al., 2018).

Unsurprisingly, temporal cues have been usefully employed to experimentally study SoA, as in the case of the Intentional Binding paradigm. Intentional Binding takes advantage of a temporal delay elapsing between an action and its sensory consequence (Engbert et al., 2007), and it stands out as the most widely used paradigm for investigating implicit SoA in the existing literature (for a critical view on its validity, see Suzuki et al., 2019). After a voluntary, intentional action (e.g., a key press) which generates an outcome after a short delay (e.g., a sound), participants report the time of the action or its outcome. Results typically show a reduced temporal estimate of the interval between the action and its outcome for voluntary actions compared with involuntary movements (Haggard et al., 2002). This compressed estimate is explained by delayed perception of the action execution as well as anticipated perception of outcome onset. On the contrary, when participants perform an action involuntarily – by having body parts passively moved – this temporal compression is not observed (Haggard et al., 2002; Engbert et al., 2008). Authors proposed that this discrepancy likely reveals the cognitive mechanisms related to SoA; in particular, the more people feel in control of an action, the stronger the temporal link between the action and its outcome (Poonian and Cunnington, 2013; Imaizumi and Tanno, 2019; Suzuki et al., 2019; Caspar et al., 2020; Ciardo et al., 2020).

Interestingly, studies have shown that pressing a button eliciting a tone and watching another person performing the same action led to similar perceived shortenings of the action-outcome interval, compared with a closely matched control stimulus (Poonian and Cunnington, 2013). These results suggest that similar processes are implemented to make causal attributions of sensory outcomes to our own actions and to others’ actions. The link between our own actions and others’ actions recalls the “motor resonance” mechanism, which refers to the activation of motor neurons when watching actions performed by other people (Avenanti et al., 2007; Uithol et al., 2011; Spaccasassi et al., 2022), supporting action understanding and imitation (di Pellegrino et al., 1992; Rizzolatti et al., 2006).

This self-other overlap goes beyond the motor domain; for instance, understanding others’ thoughts relies on the ability to understand our own thoughts (Carruthers, 1996; Waytz and Mitchell, 2011). This higher level “simulation” mechanism is well-documented in the moral cognition field: the greater the development of moral reasoning capabilities, the stronger the ability to understand the intentionality of others’ actions (Buon et al., 2013). Despite the growing interest in the scientific investigation of moral cognition (for a review, see Greene, 2015), as well as SoA (Moore et al., 2009; Moore and Haggard, 2010; Beck et al., 2017; Lush et al., 2017; Caspar et al., 2020; Ciardo et al., 2020), to date, evidence of their relation is meager. To fill this gap, here, we combined two behavioral tasks addressing moral cognition and SoA. We asked participants to perform an online revised version of the Empathy for Pain task (Decety et al., 2011) by rating, along different axes, harmful and neutral scenarios where two characters interact. In the Intentional Harm scenario, an offender deliberately inflicts pain on a victim. In the Accidental scenario, the agent accidentally provokes harm to the victim. In the Neutral scenario, the two characters’ interaction is emotionally neutral and no harm occurs. We also asked participants to perform the Intentional Binding task online to assess their SoA (Imaizumi and Tanno, 2019). If people with a stronger sense of responsibility for their own actions tend to ascribe more responsibility to the agents of observed actions, we hypothesized that they would attribute more intentionality to others, leading to harsher moral evaluations (and, possibly, a stronger desire to punish the agent) when the outcomes of the observed actions are negative, as in the case of scenarios depicting harmful interactions.

## 2. Materials and methods

### 2.1. Participants

70 participants (29 males, 60 right-handed, mean age 25.4 years, standard deviation 4.82, range: 19–45) participated in this study. Sample size was determined through a power analysis conducted using the “*pwr*” package in R (Team, 2021), with power (1–β) = 0.8, α = 0.05 and an expected effect size of 0.3, yielding a required sample size of 67 individuals. All participants self-reported having normal or corrected-to-normal vision and a normal sense of hearing, thus meeting the criteria for online participation. Most of the volunteers were students at the University of Bologna, and all gave their consent online to participate in the experiment. They did not receive any reimbursement or debriefing. This research was performed in accordance with the Declaration of Helsinki and it was approved by the Bioethical Committee of the University of Bologna (Protocol Number 284744).

### 2.2. Procedure

#### 2.2.1. Intentional binding task

**Figure 1:**
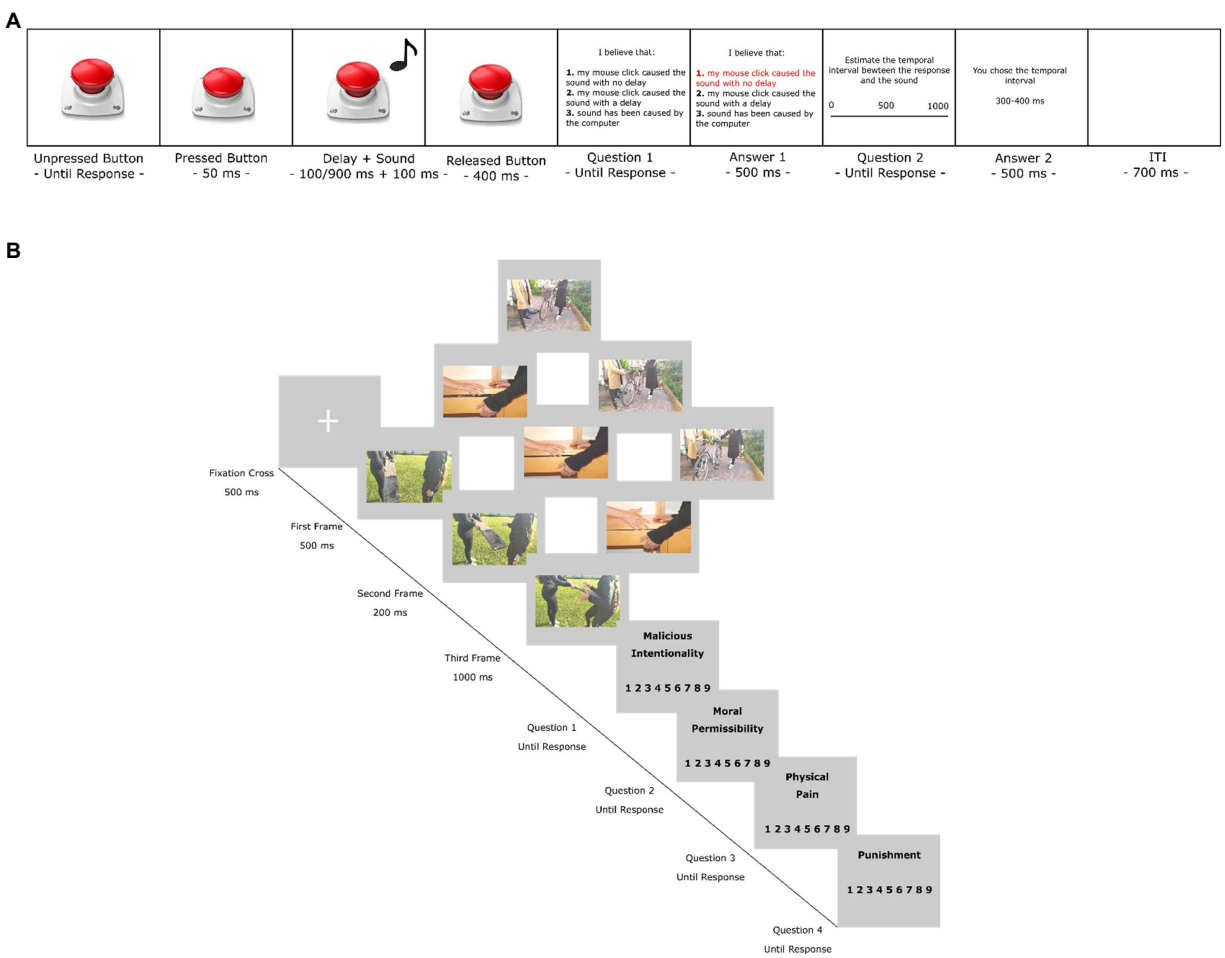
Experimental Paradigms. **(A)** Intentional Binding task: A first screen with the image of an unpressed button located at the center of the monitor was presented until a response was made (Action condition) or it remained on the screen for 700 ms (Control condition). In the Action condition, participants were asked to click with the mouse to press the button, while in the Control condition, the button was pressed automatically. As soon as the response was made by clicking the mouse (Action condition) or automatically triggered (Control condition), the button on the screen was depressed for 50 ms. Then, the button on the screen returned to an unpressed state and a sound was emitted for 100 ms after a random delay (100–900 ms). The button remained visible on the screen for another 400 ms. After that, participants were asked to respond to two questions investigating, explicit and implicit SoA, respectively. A 500 ms long visual feedback screen confirmed their choices. The interval between trials lasted 700 ms. **(B)** Moral Cognition task: Each trial started with a fixation cross (500 ms) followed by the videoclip (first frame – 500 ms, second frame – 200 ms, third frame – 1000 ms). Then, the four questions – Physical Pain, Malicious Intentionality, Moral Permissibility, Punishment – appeared on the screen and remained visible until the participant’s response. This was followed by visual feedback lasting 2000 ms. Each trial finished with an empty screen lasting 1000 ms. Please note that the first three frames of the scenario depict Intentional Harm, the second ones Accidental Harm and the third the Neutral scenario.

Participants were instructed to seat in front of their personal computer and keep a 40 cm distance from the screen throughout the entire experiment. In the Action condition, each trial started with a screen displaying an image representing an unpressed button placed at the center of the monitor. Participants were invited to press the mouse button whenever they wanted to press the virtual button on the screen. Following the button press, the image changed into a pressed button and stayed on the screen for 50 ms whereupon it returned to its initial unpressed status. A sound was delivered for 100 ms after one out of five randomly chosen temporal delays (100, 300, 500, 700, or 900 ms), while the unpressed button remained visible on the screen for 400 ms. Participants were then invited to respond to the following question by selecting one of three possible answers by pressing the corresponding number on the keyboard: (i) *I believe that: 1. The sound was caused by my mouse click without any delay; 2. The sound was caused by my mouse click with some delay; 3. The sound has been caused by the computer.* Moreover, participants were asked to (ii) *Estimate the temporal interval between the response and the sound* by clicking with the mouse in a specific position on a horizontal black bar representing the temporal interval between 0 and 1,000 ms (Figure 1A). Then, a feedback message lasting 500 ms was presented while the chosen option was highlighted in red (Question 1) and the estimated temporal interval was displayed (Question 2). In the Control trials, instead, participants did not have to perform any action. Therefore, after the first screen with the unpressed button lasting 700 ms, the button was pressed automatically (in line with Poonian and Cunnington, 2013) and remained visible in the released status for 400 ms. Afterwards, participants were required to respond to the temporal estimation question, as described above.

Each condition was repeated 14 times, for a total of 140 trials divided into two blocks (70 Action trials, 70 Control trials). The presentation order of Action and Control blocks was counterbalanced across participants. Before performing the task, participants were asked to complete a training phase consisting of 5 trials for each condition. Overall, the task lasted approximately 20 min. Prior work supports the validity of on-line versions of the Intentional Binding paradigm (Galang et al., 2021).

#### 2.2.2. Moral cognition task

At the end of the Intentional Binding task, participants were invited to watch 36 videoclips composed of three frames each for a total of 108 images (Figure 1B). Some of these videoclips were taken from Decety and Cacioppo (2012) and Baez et al. (2012, 2013, 2014), while the remainder were created in our laboratory. These videoclips were accurately matched based on the ratings of a pilot study conducted with 40 participants (see Supplementary material for further details). In each videoclip, two characters were depicted: an active character performing an action (the agent) and a passive character/victim. The agent performed an action that could result in either a neutral outcome, with no consequences for the passive character, or in a negative outcome consisting of harm to the victim; moreover, harmful actions could appear to be either intentional (i.e., stemming from the agent’s negative belief that the action will cause harm) or accidental, resulting in three types of scenarios: Intentional Harm (negative outcome/negative intention), Accidental Harm (negative outcome/neutral intention), and Neutral (neutral outcome/neutral intention). Given that the actors’ faces were not visible, participants could understand the victim’s suffering and the agent’s intention from their body expressions and postures.

Each trial started with a white fixation cross lasting 300 ms, followed by the first frame (500 ms), second frame (200 ms), and third frame (1,000 ms). At the end of each videoclip, they were asked to perform a modified version of the Empathy for Pain task (Decety et al., 2011; Baez et al., 2012, 2013, 2014; Couto et al., 2013). In the four critical questions, participants rated on a 9-point Likert scale (1 = not at all, 9 = completely) the degree to which they thought: (1) the passive character felt physical pain as a result of the protagonist’s action (Physical Pain); (2) the protagonist’s intention was malicious (Malicious Intention); (3) the protagonist’s action was morally permissible (Moral Permissibility); (4) the protagonist was worthy of punishment (Punishment). The order of trials was randomized across participants, while question order was fixed in each trial. The whole task lasted about 10 min.

Both of the tasks were programmed using OpenSesame software 3 (Mathôt et al., 2012) running on the MindProbe server powered by Jatos.^1^

### 2.3. Data analysis

Behavioral data from the Intentional Binding task were analyzed as follows: for each participant, temporal estimation trials with response times exceeding 3 standard deviations (SD) from the mean were discarded (0.38%). Then, the delay estimation error (EE) was computed by subtracting the actual interval from the estimated response (a negative value indicates underestimation, while a positive one indicates overestimation). Using the EE values, we carried out an Analysis of Variance (ANOVA) with the within-subject factors Time Interval (100, 300, 500, 700, 900) and Condition (Action, Control). Another ANOVA was also performed with the explicit Agency Ratings collected exclusively in the Action condition with the within-subject factor Time Interval (100, 300, 500, 700, and 900). In line with Poonian and Cunnington (2013), we computed the Intentional Binding index by subtracting EE values in the Control condition from those measured in the Action condition: the lower the Intentional Binding index, the stronger the time compression effect. To test the relation between implicit and explicit SoA, we carried out a correlation analysis on Intentional Binding and Agency Ratings.

For the Moral Cognition task, we first discarded values exceeding 3 SD from the group mean relative to a specific rating (0.95%). For each subscale, we carried out a separate ANOVA with the within-subject factor Scenario (Intentional, Accidental, and Neutral).

Lastly, we performed correlational analyses by computing Pearson’s correlations (r) between SoA indices (EE, Intentional Binding and Agency Ratings) and rating scales (Physical Pain, Malicious Intentionality, Moral Permissibility and Punishment) separately for each moral scenario (see Supplementary material for further analyses). When small violations of normality were detected using a Shapiro–Wilk test, we reported Spearman’s rank coefficient (rho). Frequentist correlation analyses were complemented by their Bayesian counterparts.

All frequentist analyses were performed with R software 3.6.2 (Team, 2021) using Jamovi 2.3.12 (Şahin and Aybek, 2019). The level of significance was set at *p* < 0.05. Bonferroni corrections were used for post-hoc multiple comparisons. Effect sizes were estimated by computing partial-eta^2^ (*η_p_*^2^). Bayesian analyses were performed with Jasp 0.13.1.0 (Wagenmakers et al., 2018) using default values.

## 3. Results

### 3.1. Intentional binding task

**Figure 2:**
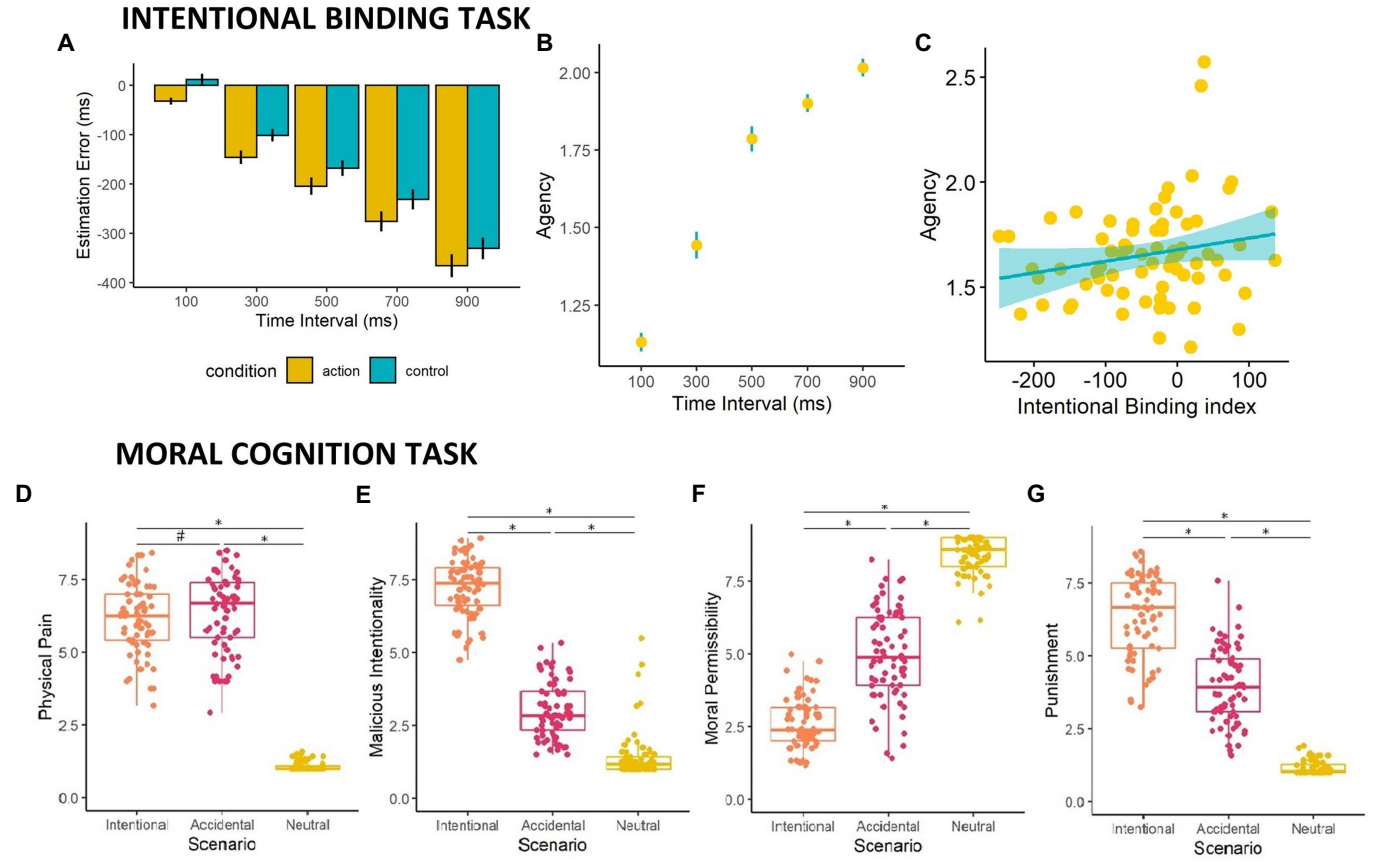
Intentional Binding Task & Moral Cognition Task Results. **(A)** The implicit measure of intentional binding (Estimation Error; y-axis) is presented for the different time intervals (x-axis). **(B)** The explicit measure of agency (Agency Rating; y-axis) is plotted in relation to the different time Intervals (x-axis). **(C)** Scatter plot showing the correlation between explicit agency (y-axis) and the Intentional Binding index (x-axis). **(D)** Box plot showing physical pain ratings. **(E)** Box plot showing malicious intentionality ratings. **(F)** Box plot showing moral permissibility rating. **(G)** Box plot showing punishment ratings. For each graph of the Moral Cognition task **(D–G)**, the x-axis represents the three different scenarios (Intentional, Accidental, Neutral) while the y-axis depicts the specific rating score. Significance levels are shown by hashes and asterisks (^#^*p* = 0.05, ^*^p < 0.001).

The Time Interval x Condition ANOVA on EE revealed a significant main effect of Time Interval (F_4,276_ = 170.40; *p* < 0.001, *η_p_*^2^ = 0.76) with each delay significantly different from the others (all *p* < 0.001), showing that participants were less accurate when estimating longer delays compared to shorter ones. Moreover, we observed a main effect of Condition (F_1,69_ = 15.54; *p* < 0.001, *η_p_*^2^ = 0.012) showing that temporal compression was overall greater in Action trials (M ± SD = −205 ± 118 ms) relative to Control trials (M ± SD = −164 ± 116 ms; Figure 2A). The two-way interaction between Time Interval and Condition did not reach significance (*p* = 0.93).

The one-way ANOVA on the Agency Ratings revealed a significant effect of Time Interval (F_4,276_ = 247, *p* < 0.001, *η_p_*^2^ = 0.561). Post-hoc tests showed that Agency Ratings were significantly different across all time intervals (all *p* < 0.001), with stronger subjective feelings of agency at shorter time intervals^2^ (Figure 2B).

Correlation analyses between the Intentional Binding Index and Agency Ratings revealed a significant positive relationship (*r* = 0.204, *p* = 0.045), with stronger explicit agency ratings linked to stronger intentional binding effects (Figure 2C).

### 3.2. Moral cognition task

Figure 2 also shows participants’ ratings of the three moral scenarios. The ANOVA on pain ratings was significant (F_2,136_ = 1,021, *p* < 0.001, *η_p_*^2^ = 0.850; Figure 2D). Participants rated the perceived pain of the victim marginally higher in Accidental scenarios (M ± SD: 6.38 ± 1.34) compared to Intentional (6.16 ± 1.19; *p* = 0.046) and Neutral scenarios (1.09 ± 0.17; *p* < 0.001), which in turn significantly differed from each other (*p* < 0.001).

The ANOVA on intentionality ratings was significant (F_2,130_ = 912, *p* < 0.001, *η_p_*^2^ = 0.873; Figure 2E) showing a higher attribution of malicious intentionality for Intentional scenarios (7.24 ± 0.99) compared to Accidental (3.04 ± 0.97; *p* < 0.001) and Neutral scenarios (1.43 ± 0.86; *p* < 0.001), which in turn significantly differed from each other (p < 0.001).

The ANOVA on moral permissibility ratings was also significant (F_2,136_ = 739, *p* < 0.001, *η_p_*^2^ = 0.828; Figure 2F). Participants rated the action less morally permissible in Intentional scenarios (2.59 ± 0.87) relative to Accidental (4.98 ± 1.56; *p* < 0.001) and Neutral scenarios (8.41 ± 0.66; *p* < 0.001), which in turn significantly differed from each other (p < 0.001).

Lastly, the ANOVA on punishment (F_2,134_ = 581, *p* < 0.001, *η_p_*^2^ = 0.794; Figure 2G) showed greater willingness to punish the agent in Intentional scenarios (6.40 ± 1.38) compared to Accidental (3.98 ± 1.27; *p* < 0.001) and Neutral scenarios (1.16 ± 0.23; *p* < 0.001), which in turn significantly differed from each other (*p* < 0.001). Overall, these results suggest that participants correctly evaluated the scenarios.

### Correlation analysis

**Figure 3:**
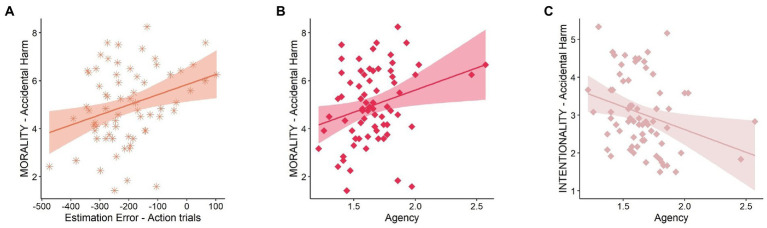
Correlation Analysis Results. **(A)** Scatter plot showing the correlation between moral permissibility ratings of Accidental Harm scenarios (y-axis) and the estimation error averaged across all the time intervals in Action trials only (x-axis). **(B)** Scatter plot showing the correlation between moral permissibility ratings of Accidental Harm scenarios (y-axis) and Explicit Agency (x-axis). **(C)** Scatter plot showing the correlation between malicious intentionality ratings of Accidental Harm scenarios (y-axis) and Explicit Agency (x-axis).

We observed a positive correlation between averaged EE for Action trials and moral permissibility ratings of Accidental Harm scenarios (*r* = 0.32, *p* = 0.004, BF_+0_ = 10.321); this indicates that those participants who reported stricter moral judgments of accidental harms also showed more temporal compression between their own actions and the subsequent sounds, which could be considered an implicit measure of SoA (Figure 3A). A positive correlation was also found between explicit agency ratings and the moral permissibility of Accidental Harm (rho = 0.265, *p* = 0.013, BF_+0_ = 4.243), indicating stricter moral judgments of accidental injuries for participants with stronger explicit SoA (Figure 3B). Finally, we observed another correlation between explicit agency and malicious intentionality ratings of Accidental Harm (rho = −0.326, *p* = 0.003, BF_+0_ = 16.895), suggesting a higher attribution of malicious intentionality in cases of accidental injury for participants with higher explicit SoA (Figure 3C). All the significant correlations survived a correction for multiple comparisons (all *p* < 0.05). No other significant correlations found (all *p* > 0.05; see Supplementary material for confirmatory analyses).

## 4. Discussion

In the present study, we sought to investigate whether there is a correspondence between the Sense of Agency (SoA) and the way we morally evaluate others’ actions. SoA was measured through the Intentional Binding task (Imaizumi and Tanno, 2019), which allowed us to measure, both implicitly and explicitly, the subjective feeling of being in control of one’s own actions by means of temporal estimation and agency ratings, respectively. In the Moral Cognition task, participants evaluated, along four different axes, the intentionally harmful, accidentally harmful and neutral actions performed by others. Our main results demonstrate a consistent relation between SoA and morality judgments, since we found two significant correlations between the way we judge the moral permissibility of Accidental Harm scenarios and SoA (both its explicit and implicit components). Specifically, the greater the SoA, the stricter the moral judgements of agents in these scenarios. In addition, and only for explicit SoA, we observed a correlation with Malicious Intentionality ratings of the same scenarios: the stronger the SoA, the higher the attribution of malicious intentionality to the agent in Accidental scenarios. This pattern was specific to Accidental Harm as it was not found with the other two scenarios.

Concerning Intentional Binding, we replicated the well-established underestimation effect (Poonian and Cunnington, 2013; Imaizumi and Tanno, 2019), wherein people tend to show greater underestimation of the temporal interval between their action and its associated consequence, compared to control conditions. We confirmed this result by showing greater temporal compression of the action-outcome interval in the condition where participant’s own actions produced the outcomes, compared to the passing viewing condition. Our data suggest that Intentional Binding is comparable across temporal delays; we did not find a significant interaction between the Time Interval and Condition factors. This result is perfectly in line with previous studies adopting the same experimental paradigm (Imaizumi and Tanno, 2019; for different results see Zapparoli et al., 2020).

Regarding explicit SoA, we observed, instead, that it decreased at increasing time intervals: the shorter the delay between the action and the outcome, the stronger the explicit SoA (in line with Poonian and Cunnington, 2013; Wen et al., 2015; Imaizumi and Tanno, 2019). Importantly, the intentional binding index also correlated with the averaged explicit agency ratings, with shorter temporal estimations associated with higher explicit SoA (as reported also in Imaizumi and Tanno, 2019; Galang et al., 2021; for different results see Moore et al., 2012; Dewey and Knoblich, 2014; Saito et al., 2015; Wen et al., 2015; Ma et al., 2021). In their multifactorial two-step model, Synofzik et al. (2008) argued that bottom-up mechanisms account for the feeling of agency (implicit SoA), which is further processed through top-down mechanisms to form an explicit attribution of agency (explicit SoA). Following this line of thought, it seems that, in our case, the bottom-up processing of perceptual output and the top-down influence of cognitive factors showed similar patterns of effects.

Concerning the Moral Cognition task, we found that participants relied on both agent intention and action outcome when judging the morality of the scenarios (Young and Saxe, 2009; Baez et al., 2014; Gan et al., 2016). Indeed, intentional pain scenarios were judged more severely than accidental pain scenarios; moreover, despite sharing the same neutral intention, accidental harm scenarios were considered less morally permissible than neutral scenarios. These effects likely emerged due to the different outcomes of the two types of actions: victim’s suffering in the former, but not in the latter. Dual-system approaches explain the fundamental properties of human moral judgments by contrasting “emotional” and “rational/cognitive” processes (Cushman, 2013). Emotional processes are involved in the affective reaction to the injury experienced by the victim (action outcome), while the cognitive processes are involved in inferring the agent’s mental state (agent intention). Because Accidental Harm and Neutral actions share the same neutral intention (cognitive processes), the moral permissibility of the scenarios mostly depends on emotional processes, which are recruited when watching someone’s suffering (Accidental Harm) compared to a situation where there is no harm for anybody (Neutral scenarios).

Crucially, when comparing the results of the Intentional Binding and Moral Cognition tasks, we found two significant correlations – also strongly supported by Bayesian analyses – between implicit and explicit SoA indices and moral judgments of Accidental Harm scenarios. Specifically, the stronger the SoA – meaning a greater temporal underestimation effect for Action trials on the implicit intentional binding measure – the stricter the moral judgment. Moreover, for the explicit measure of agency only, and in line with our previous finding, we also observed a significant relation with the intentionality rating of the same scenarios: the stronger the agency, the more malicious the intentionality was evaluated to be. This self-other overlap is reminiscent of the “simulation theory” proposals (Carruthers, 1996; Waytz and Mitchell, 2011), which posit that perceivers can use knowledge about themselves to infer the mental states of others. In this vein, feeling more responsible for our own actions would predict a similar tendency when judging the actions of others.

It is not surprising that all these correlations emerged only with Accidental Harm scenarios. Indeed, as recently proposed by Hirschfeld-Kroen et al. (2021), Accidental Harm scenarios are an important context for broadening our understanding of the relation between agency and moral judgments thanks to the incongruence between the agent’s intention, which is neutral, and the action’s outcome, which is instead negative (Young and Saxe, 2009). Indeed, for judging these scenarios, people must inhibit a salient and prepotent representation (i.e., the observed outcome for the victim) and assign more weight to a less immediate alternative (i.e., the intention of the agent; Young and Saxe, 2009; Margoni and Surian, 2016, 2021; Baez et al., 2018; Schwartz et al., 2021). Thus, the pattern of correlations that we observed may suggest that people with stronger SoA fail to inhibit outcome/victim-based emotional processes. Future research is needed to understand whether stronger SoA is associated with a more general reduced ability to inhibit irrelevant information. Moreover, future studies over larger samples are needed to replicate the results of the present exploratory study.

Our results appear to agree with another study investigating the relation between SoA and morality: Moretto et al. (2011) found enhanced SoA in moral compared to non-moral contexts. Indeed, participants exhibited stronger picture-effects binding when the picture represented a moral dilemma compared with a purely economic dilemma. This suggests that agency is perceived differently in morally salient events relative to other events. Furthermore, our data are also in line with those reported by Lepron et al. (2015), where being held responsible for third-person pain caused an enhanced empathic response, suggesting that SoA may play a role in regulating empathy and, consequently, moral conduct. Importantly, the results of our research - the more you master your own actions, the more rigorous your moral judgment is - add to the previous literature suggesting that our sense of responsibility for our own actions goes hand in hand with our moral rigor for others’ actions. We could conclude by saying that a “*responsible brain*” is also a “*moral brain*.”

## Data availability statement

The raw data supporting the conclusions of this article will be made available by the authors, without undue reservation.

## Ethics statement

The studies involving human participants were reviewed and approved by Local ethical committee of the University of Bologna (Protocol Number 284744). Written informed consent was not provided because of the online data collection procedure where inform consent was inherent to the participation to the experiment.

## Author contributions

CS was responsible for the conceptualization, experimental design, data analyses, and manuscript writing for this project. KC was responsible for the experimental design, data collection and analysis, and manuscript writing. SP was responsible for the experimental design, data collection, and manuscript revision. AA was responsible for the conceptualization, experimental design, resource acquisition, and manuscript revision. All authors contributed to the article and approved the submitted version.

## Funding

This work was financially supported by research grants from the Bial Foundation (347/18 and 304/2022), Fondazione del Monte di Bologna e Ravenna (1402bis/2021), and the Ministero dell’Istruzione, dell’Università e della Ricerca (2017N7WCLP) awarded to AA.

## Conflict of interest

The authors declare that the research was conducted in the absence of any commercial or financial relationships that could be construed as a potential conflict of interest.

## Publisher’s note

All claims expressed in this article are solely those of the authors and do not necessarily represent those of their affiliated organizations, or those of the publisher, the editors and the reviewers. Any product that may be evaluated in this article, or claim that may be made by its manufacturer, is not guaranteed or endorsed by the publisher.

## Footnotes

^1^ http://mindprobe.eu

^2^Please note that lower scores on the agency rating scale indicate stronger subjective feelings of agency while higher scores indicate weaker agency.
