# Supplementary material for: Sense of agency predicts severity of moral judgments
Source: Front Psychol. 2023 Feb 2;13:1070742. doi: 10.3389/fpsyg.2022.1070742 (PMC9932714; doi:10.3389/fpsyg.2022.1070742)
Supplement: Supplementary file 1 [file Data_Sheet_1.docx]

**Sense of Agency Predicts Severity of Moral Judgments**

**Supplementary Material**

Chiara Spaccasassi, Kamela Cenka, Stella Petkovic, Alessio Avenanti

**Supplementary Method**

**Validation phase.** In a pre-experimental validation session, a group of participants (N = 40, 23 females, mean age ± sd = 24.95 ± 2.24 years, range = 21-30 years) assessed 90 video clips using a 9-point Likert scale. Participants had to rate: (*i*) physical pain, “*How much physical pain did the victim feel in the scene you just observed?*”; (*ii*) moral permissibility, “*How morally permissible was the action in the scene you just observed?*”; (*iii*) malicious intentionality, “*How bad was the action intention of the agent in the scene you just observed?*”; (*iv*) arousal “*How strong was the emotion you felt for the scene you just observed?*”; (*v*) movement “*How much movement was there in the scene you just observed?*”; and (*vi*) visual background “*How complex was the visual background in the scene you just observed?*”. After presentation of the video clip at the center of the screen, participants had to provide their answers by choosing one out of 9 alternatives (from 1 “*not* *at all*” to 9 “*completely*”, where the central score was the neutral point). Each video in the experiment was composed of three photos presented in rapid succession (first frame 500 ms, second frame 200 ms, third frame 1000 ms) lasting 1700 ms overall. These video clips were partly retrieved from Decety & Cacioppo (2012) and Baez et al. (2012, 2013, 2014), while the remaining set was created within our laboratory. They included 30 accidental harm, 30 intentional harm and 30 neutral video clips, for a total of 270 images. This procedure allowed us to select the final 36 video clips (12 accidental harm, 12 intentional harm, 12 neutral scenes) subsequently used in the main experiment (see Main Experiment for further details). First, we inspected the descriptive statistics and chose those videos characterized by the most appropriate scores on all six scales. Then, we conducted a confirmatory repeated measures ANOVAs with Scenario (Intentional, Accidental, Neutral) as the within-subjects factor, analyzing the scores on each scale separately. Post-hoc analyses were conducted using a Bonferroni correction. Figure S1 shows participants’ ratings of the selected scenarios along the six evaluated dimensions.

**Physical Pain.** We observed a significant main effect of Scenario (F_2,78_ = 463; p < 0.001). Post-hoc analysis (Bonferroni) revealed that scores for neutral scenes were lower than those for both accidental and intentional scenes (both p < 0.001), which in turn did not differ from one another (p = 0.852) (accidental: M ± DS = 6.12 ± 1.45; intentional: M ± DS = 5.92 ± 1.42; neutral: M ± DS = 1.09 ± 0.18).

**Moral Permissibility.** We observed a significant main effect of Scenario (F_2,78_=428; p<0.001). Post-hoc analysis revealed that all three scenes significantly differed from each other (all p<0.001), with the lowest scores observed for intentional harm, followed by accidental harm and then neutral scenes (accidental: M ± DS = 5.24 ± 1.54; intentional: M ± DS = 2.65 ± 0.98; neutral: M ± DS = 8.69 ± 0.39).

**Malicious** **Intentionality.** We observed a significant main effect of Scenario (F_2,78_=374; p<0.001), with all three scenes significantly differing from each other (all p<0.001). We found the lowest scores for neutral scenes, followed by accidental harm and then intentional harm scenes (accidental: M±DS=3.37±1.06; intentional: M±DS=7.17±0.86; neutral: M±DS=1.35±1.16).

**Arousal.** We observed a main effect of Scenario (F_2,78_=164; p<0.001): scores for neutral scenes were lower than scores for both accidental and intentional harm scenes (both p < 0.001), which in turn did not differ from one another (p=0.287) (accidental: M±DS=4.39±1.53; intentional: M±DS=4.67±1.53; neutral: M±DS=1.97±0.86).

**Movement.** A significant main effect of Scenario (F_2,78_ = 67.1; p < 0.001) showed that neutral scenes were rated lower than both accidental and intentional harm scenes (both p<0.001), which in turn did not differ from one another (p = 0.481) (accidental: M ± DS = 4.16 ± 1.05; intentional: M ± DS = 4.33 ± 1.29; neutral: M ± DS = 3.08 ± 1.13). To further control for pixel modification between the three scenes, we computed an index of pixel variability within every video clip through the R package “imager” (Barthelmè & Tschumperlè, 2019) using the function “imshift” which counts the number of displacements along the different axes. Those values were analyzed with a repeated measures ANOVA with the factor Scenario (accidental, intentional, neutral), and showed no consistent differences (F_2,22_ = 3.20; p = 0.061).

**Visual Background.** The main effect of Scenario did not reach significance (F_2,78_ = 1.18; p = 0.313), indicating that the three categories did not significantly differ in terms of visual scene complexity (accidental: M ± DS = 2.85 ± 1.00; intentional: M ± DS = 2.70 ± 1.04; neutral: M ± DS = 2.79 ± 1.15). The validation phase lasted about 45 minutes. The experiment was programmed using OpenSesame software (Mathôt et al., 2012) and was run online through the Jatos server (<https://www.jatos.org/>) via a MindProbe account (<https://mindprobe.eu/>).


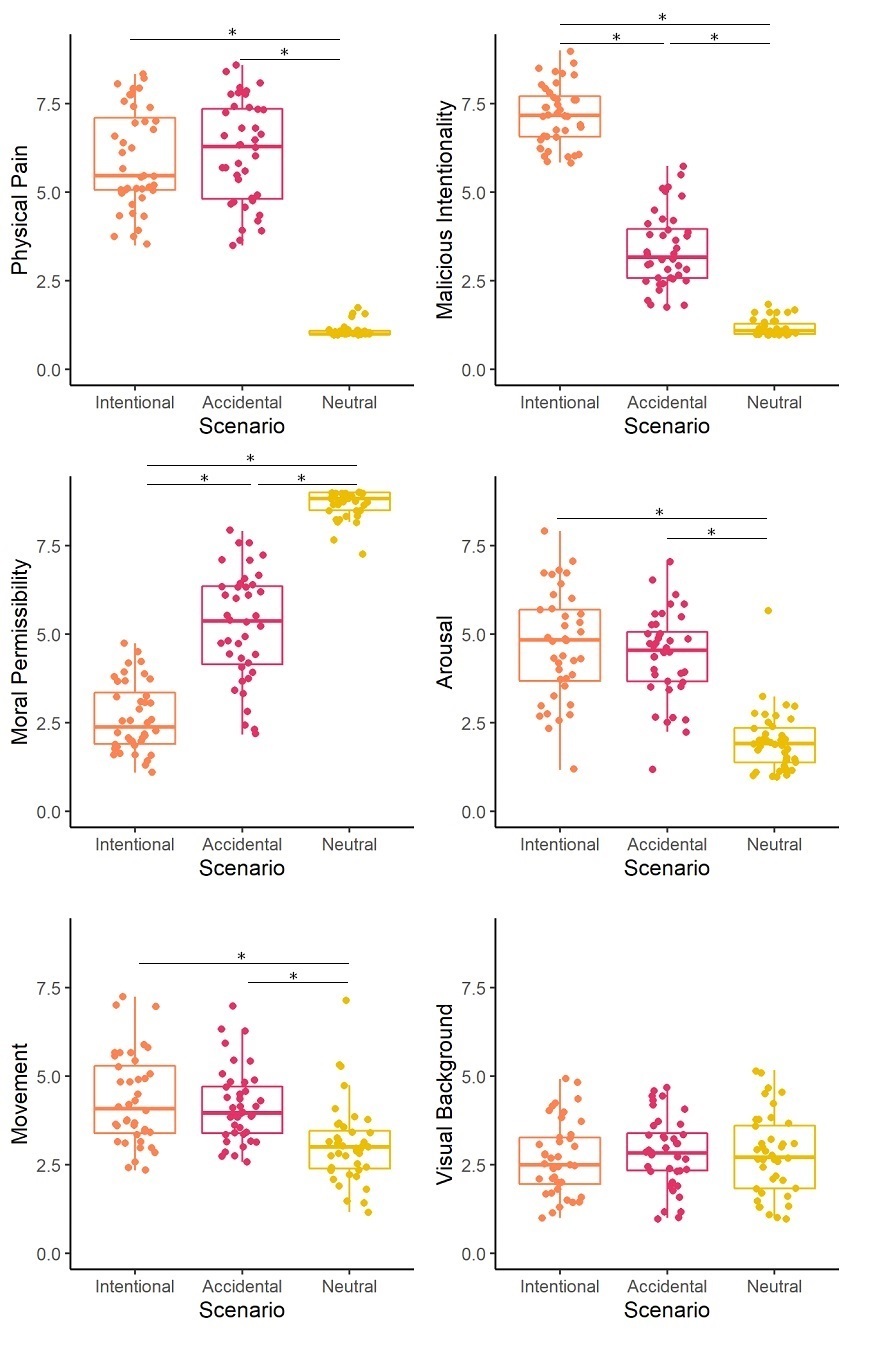


**Supplementary Figure 1.** **Results of the validation phase**. In all six graphs, the variable Scenario is represented on the x-axis while rating scores are depicted on the y-axis. Red indicates Accidental Harm scenarios, orange indicates Intentional Harm scenarios and yellow indicates Neutral scenarios. The significance level is shown by asterisks (# p = 0.05, * p < 0.001).

**Correlation Analysis.** As a further control analysis, we explored the pattern of correlations between moral cognition ratings and sense of agency (SoA) indices by computing a moral judgment score – similar to the intentional binding score– as the difference between the two critical scenarios, Intentional and Accidental Harm, divided by the Neutral video clip scores [(Intentional – Accidental)/Neutral]. We carried out correlation analyses between this index, computed for each rating scale of the Moral Cognition task, and the SoA indices (temporal estimation error, intentional binding and agency ratings).

Results confirmed the three significant correlations reported in the main text using the raw data. Indeed, we observed two negative correlations between averaged EE/agency ratings for Action trials and the moral permissibility index (EE: r = -0.241, p = 0.023, BF_+0_ = 2.049; agency ratings: rho = -0.240, p = 0.023, BF_-0_ = 2.096). This indicates that people who evaluated the moral permissibility of Intentional and Accidental Harm scenarios as almost equivalent have stronger SoA on both implicit and explicit measures. The positive correlation between agency ratings and the malicious intentionality index (rho = 0.220, p = 0.037, BF_+0_ = 2.096) suggests that people with stronger explicit SoA evaluated the malicious intentionality of Intentional and Accidental Harm scenarios similarly. Additionally, we found a negative correlation between intentional binding and the physical pain index (r = -0.300, p = 0.012, BF_10_ = 3.257), indicating that people with stronger implicit SoA tended to attribute more physical pain to the victims in the Intentional Harm scenarios than the Accidental Harm scenarios (for all the analyses, see Supplementary Figure 2). No other significant correlations were found (all ps > 0.05).


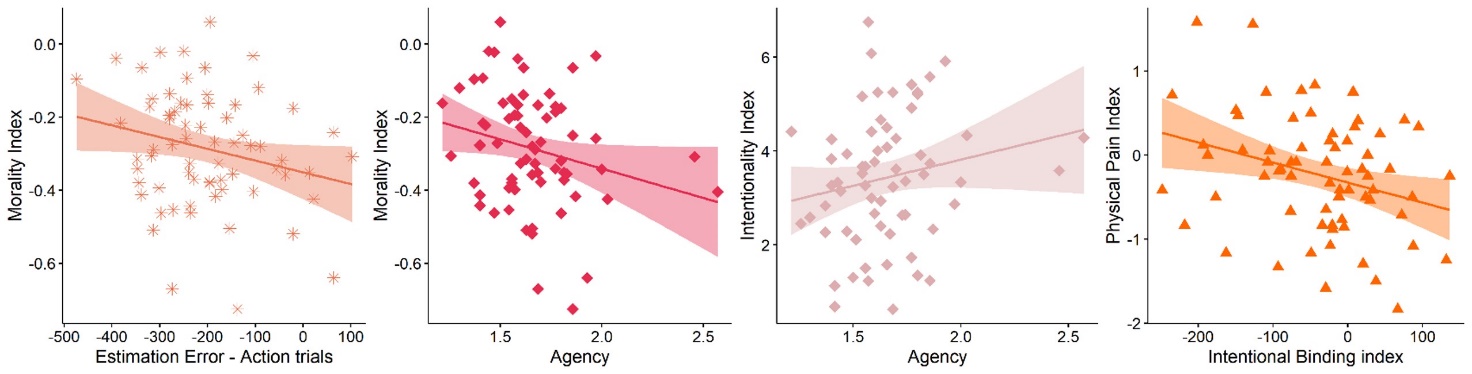


**Supplementary Figure 2.** **Results of correlation analysis**. From left to right: *(i)* scatter plot showing the correlation between the moral permissibility index (y-axis) and the estimation error averaged across all the time intervals in Action trials only (x-axis); *(ii)* scatter plot showing the correlation between the moral permissibility index (y-axis) and explicit agency ratings (x-axis); *(iii)* scatter plot showing the correlation between the malicious intentionality index (y-axis) and explicit agency ratings (x-axis); *(iv)* scatter plot showing the correlation between the physical pain index (y-axis) and the intentional binding index averaged across all the time intervals (x-axis) .

**Supplementary References**

Baez, S., Herrera, E., Villarin, L., Theil, D., Gonzalez-Gadea, M. L., Gomez, P., Mosquera, M., Huepe, D., Strejilevich, S., Vigliecca, N. S., Matthäus, F., Decety, J., Manes, F., & Ibañez, A. M. (2013). Contextual social cognition impairments in schizophrenia and bipolar disorder. PloS One, 8(3), e57664.

Baez, S., Manes, F., Huepe, D., Torralva, T., Fiorentino, N., Richter, F., Huepe-Artigas, D., Ferrari, J., Montañes, P., Reyes, P., Matallana, D., Vigliecca, N. S., Decety, J., & Ibanez, A. (2014). Primary empathy deficits in frontotemporal dementia. Frontiers in Aging Neuroscience, 6, 262.

Baez, S., Rattazzi, A., Gonzalez-Gadea, M. L., Torralva, T., Vigliecca, N. S., Decety, J., Manes, F., & Ibanez, A. (2012). Integrating intention and context: assessing social cognition in adults with Asperger syndrome. Frontiers in Human Neuroscience, 6, 302.

Barthelmé, S., & Tschumperlé, D. (2019). imager: an R package for image processing based on CImg. Journal of Open Source Software, 4(38), 1012.

Decety, J., & Cacioppo, S. (2012). The speed of morality: a high-density electrical neuroimaging study. Journal of Neurophysiology, 108(11), 3068-3072.

Mathôt, S., Schreij, D., & Theeuwes, J. (2012). OpenSesame: An open-source, graphical experiment builder for the social sciences. Behavior Research Methods, 44(2), 314-324.
